# Fibrinolysinoma after Direct Injection of Fibrinolysin for Treatment of Nasal Tip Swelling: A Case Report

**Published:** 2012-01

**Authors:** Abdoljalil Kalantar Hormozi, Seyed Reza Rafei

**Affiliations:** 1 Department of Plastic and Reconstructive Surgery, Shahid Beheshti University of Medical Sciences, Tehran, Iran.; 2 Department of Plastic and Reconstructive Surgery, Boushehr University of Medical Sciences, Boushehr, Iran.

**Keywords:** Rhinoplasty, Fibrinolysin, Polybeak deformity, Tip edema, Supratip deformity

## Abstract

Rhinoplasty is one of the most common aesthetic procedures in the world. It is the most common aesthetic surgery procedure in Iran. Achieving complete patient satisfaction is almost impossible but improving the aesthetics and function of the nose is the aim of surgery. This report describes a 34 years old woman with a large bump in the tip of the nose after three times cosmetic rhinoplasties. The first time was done 4 years before referral to our center as a reduction rhinoplasty operation. The next two subsequent surgeries were performed for revision and correcting the dorsal irregularities and supra tip bulge.

Unfortunately the supratip bulge persisted and subsequent subcutaneous injections of corticosteroids and fibrinolysin were carried out. She developed a bulbous tip nose deformity. Therefore, after a few months; she was referred to our center. Surgical exploration showed a lesion in the supratip region and histopathologic examination showed a foreign body reaction and granuloma formation.

## INTRODUCTION

Nasal tip remodeling is one of the most challenging areas in rhinoplasty.^1^ Many of revisions or secondary rhinoplastic deformities are because of the need for correction of nasal tip deformities. Nasal tip consists of important structures like alar cartilages, nostril lobules, soft triangle, columella and supratip area cartilages, nostril lobules, soft triangle, columella and supratip area.^2^ On the other hand, postoperative nasal tip swelling is another controversial discussion that has not touched the end till now.

To solve this problem several procedures were recommended. Long time taping, injection of corticosteroids, application of electromagnetic devices, oral corticosteroids, and using NSAIDs are current advised protocols.^8^ So many routessfor delivery of a drug to tissue were introduced such as 

intralesional injection, transdermal drug delivery, etc; among them, transdermal delivery was shown to improve the therapeutic efficacy of drugs in a more precise and specific way.^2^

The major barrier to the delivery of transcutaneous drugsis skin. The stratum cornium is believed to provide the major physical barrier for most of them. Several new active-rate controlled transdermal drugs technologies (electrically based, structure–based, velocity- based, etc.) were developed and commercialized for the transdermal delivery of troublesome drugs. Drug molecules can penetrate the epithelium transcellulary or intercellulary through channelsbetween cellsor they may gain transappendageal entry through the skin appendagessuch as sebaceous,sweat gland duct. A drug to be a practical candidate for transdermal route, it must possess physicochemical properties that are associated with relatively high properties including a low molecular weight (<1000) oil and water. New active transport technologies have been developed for the transdermal delivery of transdermal drugs to overcome the limitations of chemical enhancement techniques such as molecular weight limitation in sonophoresis and iontophoresis and macroflux.^2^

## CASE REPORT

**Fig. 1:**
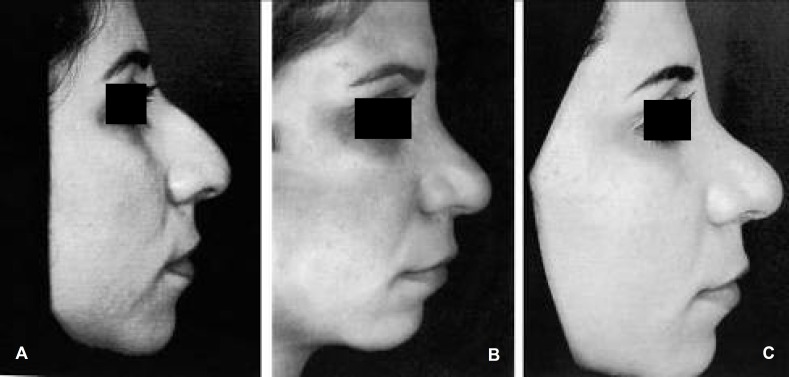
A (Lt) Preoperative profile view, B (center) Post operative profile view after first surgery, C (Rt) Post operative profile view after second surgery and augmentation by iliac bone graft.

A 34- year old woman was referred to our clinic with a few months history of a bulge in her nose tip. Four years before being referred to our center, she had undergone a cosmetic reduction rhinoplasty in another center (Figure 1A). She was unsatisfied because she had developed a large supratip fullness (Figure 1B).

Afterwards, she underwent a second surgery a year later and the nasal dorsal defect was augmented by iliac bone graft (Figure 1C). The second surgery could not fulfill the patient’s desire and she was still unhappy with the result. Therefore, she was operated for the third time and tip grafting was done treating by systemic corticosteroids and repeated using high dose triamcinolon injections.

**Fig. 2:**
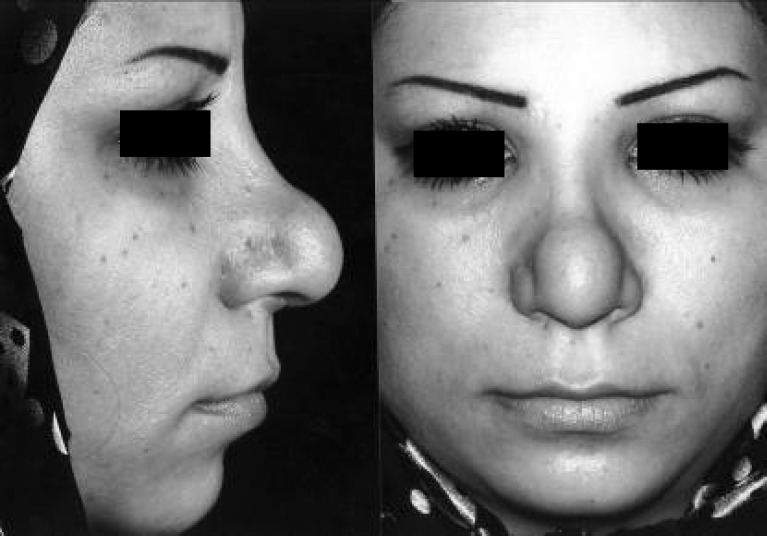
Well defined round mass in tip and supra tip after fibrinolysin injection. Left: profile view, Right: frontal view.

**Fig. 3:**
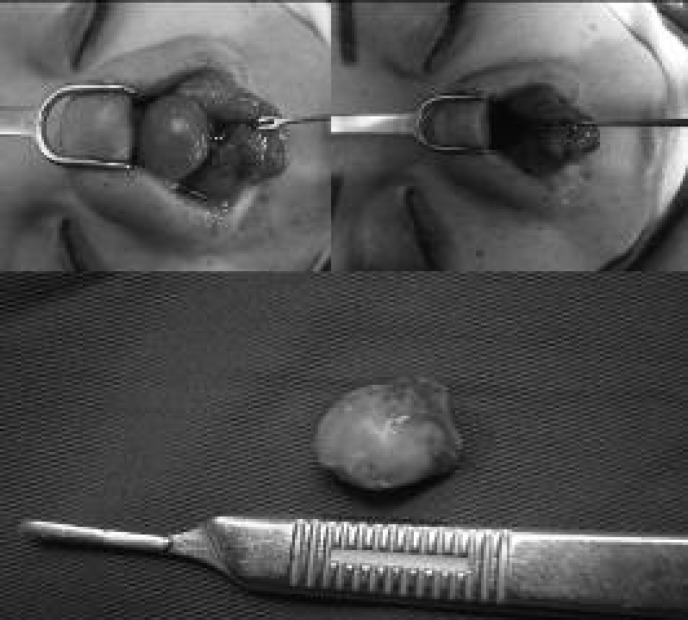
Discrete round mass was found and resected during the last operation.

**Fig. 4:**
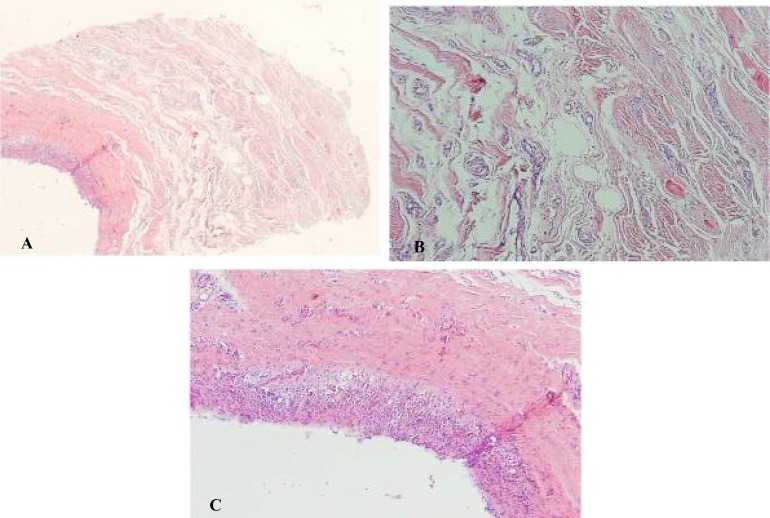
(A) Cut Section of cyst wall showes granulation tissue formation and severe stromal edema. (B) New capillary formation and severe edema and chronic inflammation. (C) Granulation tissue formation and underlying fibroblastic proliferation.

**Fig. 5:**
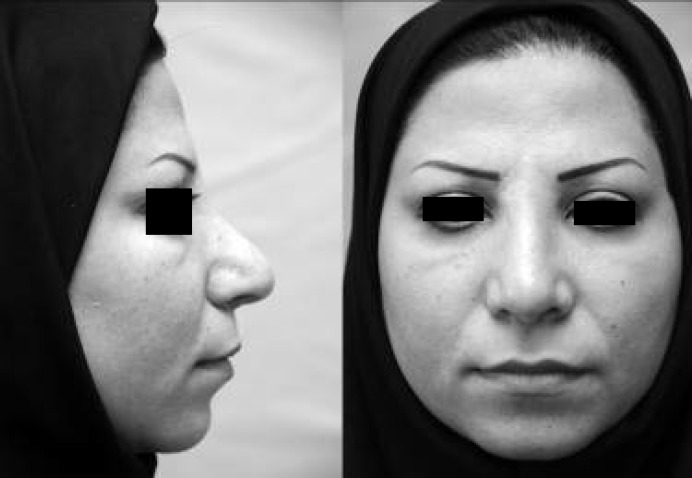
Final result of surgery after mass removal. Left: (profile view), Right (frontal view).

Since the problem was not yet resolved, the surgeon performed two subsequent injections of fibrinolysin in her supratip area. By the time, she was referred to our center. She had developed a well-defined round mass with firm consistency in the tip of her nose (Figure 2). She did not suffer from any medical problem, and the bulging seems to be the result of the fibrinolysin injection. In our center, she underwent operation for the fourth time. During an open rhinoplasty operation, a discrete round mass was found (Figure 3). The mass was delivered and the operation was terminated. Pathologic examination showed fibrosis and foreign body reaction (figure 4). The final result of surgery is demonstrated in Figure 5. 

## DISCUSSION

Rhinoplasty is the most common aesthetic surgeries in our country and thus it encounters more complications than any other aesthetic surgery.^3^ Approximatly 20% of the primary rhinoplasty patients are suitable candidate for traditional reduction rhinoplasty.^4^ Nowadays, facing a middle eastern nose, surgeons use cartilage conservation instead of previously reduction techniques. 

One of the main problems in middle eastern rhinoplasty is the poor contractile nature of thick, sebaceous skin in the supratip region. poor contractile ability of this tissue and presence of skin excess at completion of the rhinoplasry can lead to disappointing long term result and is perhaps the most difficult component to control.^5^ Supratip deformity can result from over-reduction of dorsum and radix and loss of tip support.^6^ A logical treatment of these pathological findings is dorsal nasal augmentation and improving tip support.

It seems that avoiding over resection is the best way of prevention of such problems. 

The pathomechnism, prevention and treatment of this problem was outlined clearly before.^7^ Considering the patients desire, surgeons have to try to create a balanced nose and facial configuration. Prevention of short nose and racial incongruity is the main concern in all rhinoplasty procedures, especially in middle eastern noses. Surgeon must recognize the cause of post-operative fullness in the supratip area. Sometimes, fullness is due to edema and occasionally is related to overresection of cartilage that results to a polybeak deformity.^8^

When the edema in the tip of nose has been recognized, corticosteroid injection can be a logical option. Treatment with local injection of corticosteroid is a perfectly acceptable means of reducing the edema and subsequent fibrosis if excessive edema persists at 3 to 6 weeks after surgery.^9^

Unfortunately, the unjustified injection of a locally active ointment to nasal subcutaneous tissue had resulted in unaesthetic appearance of the patient’s face. Also in case of tip edema, intralesional dilute triamecinolon can be used. In addition, fibrinolysin (Elase) is widely used ointment consisting of a combination proteolytic enzymes, fibrinolysin and desoxyribonuclease (DNAse).^10^ It promotes debridement of necrotic and purulent debris from skin ulcers but there is no indication for subcutaneous injection of topical fibrinolysin.

We believe that the best way of preventing such post-surgery complications is to recognize the pathology of nose in mainstay of rhinoptasly design. Also, selecting the right technique for rhinoplasty is an important factor. However, in case of complication; treatment must be based on the exact pathologic diagnosis of the deformity. Although corticosteroids can resolve the edema, while they were not effective in our case as her problem was over-resection of a dorsal nose.

In case of supra-tip deformity and fullness resulting from nasal dorsal over-resection dorsal augmentation is the best procedure. Avoidance of use of unusual medicines or unjustified surgical techniques and not being harmful for the patient should be our dictum.

## CONFLICT OF INTEREST

The authors declare no conflict of interest.
